# Effects of Dietary Forage and Calf Starter Diet on Ruminal pH and Bacteria in Holstein Calves during Weaning Transition

**DOI:** 10.3389/fmicb.2016.01575

**Published:** 2016-10-21

**Authors:** Yo-Han Kim, Rie Nagata, Natsuki Ohtani, Toshihiro Ichijo, Kentaro Ikuta, Shigeru Sato

**Affiliations:** ^1^United Graduate School of Veterinary Science, Gifu UniversityGifu, Japan; ^2^Cooperative Department of Veterinary Medicine, Faculty of Agriculture, Iwate UniversityMorioka, Japan; ^3^Awaji Agricultural Technology CenterMinami-Awaji, Japan

**Keywords:** ruminal pH, ruminal bacteria, calf weaning, 454 pyrosequencing, forage

## Abstract

We investigated the relationship between ruminal pH and bacteria in calves fed calf starter with and without forage during weaning transition. First, 16 Holstein bull calves were obtained from dairy farms and equipped with rumen cannulas by cannulation surgery. Then, calves (73.5 ± 4.2 kg; mean ± SE) were assigned to groups fed calf starter either with forage (HAY, *n* = 8) or without forage (CON, *n* = 8), and all calves were weaned at 8 weeks of age. Ruminal pH was measured continuously, and rumen fluid samples were collected at 7, 8, 9, and 11 weeks of age, namely −1, 0, 1, and 3 weeks after weaning, respectively, to assess volatile fatty acid concentrations and bacterial DNA. The 24-h mean ruminal pH was significantly (*P* < 0.05) different between the two groups. Diurnal changes in the 1-h mean ruminal pH were observed throughout the study in the HAY group; however, they were not observed at 0 and 1 weeks after weaning in the CON group. Moreover, the HAY group had significantly (*P* < 0.05) higher proportions of acetate and butyrate and lower proportion of propionate, and significantly (*P* < 0.05) lower ruminal acetate-to-propionate ratios were observed in the CON group. The ruminal bacterial diversity indices decreased after −1 week in both groups and increased at 0 and 1 weeks after weaning in the HAY and CON groups, respectively. From the 454 pyrosequencing analysis, significant differences (*P* < 0.05) were observed in the relative abundance of several phyla (*Bacteroidetes, Actinobacteria*, and *Tenericutes*) and one genus (*Prevotella*) between the two groups. From quantitative real-time PCR analysis, the HAY group had the higher copy numbers of cellulolytic bacteria (*Ruminococcus flavefaciens* and *Ruminococcus albus*) compared with the CON group. This study demonstrated that feeding of dietary forage alleviates subacute ruminal acidosis due to diurnal changes in ruminal pH. Furthermore, changes in ruminal pH affect the ruminal bacterial diversity and relative abundance, and these changes might have influenced the establishment of fermentative ruminal functions during weaning transition.

## Introduction

Weaning transition is defined as the period of transition from liquid to solid feed consumption, which is critical for the development of an active and functional rumen (National Research Council, 2001). For example, calves fed starch sources during weaning transition exhibit increased volatile fatty acid (VFA) and lactic acid production, which decreases ruminal pH (Laarman and Oba, 2011). Increased VFA production, especially butyrate, via solid feed fermentation in the developing rumen is responsible for functional ruminal epithelial tissue development (Sander et al., 1959). Conversely, excessive amounts of rapidly fermentable carbohydrates in feed can cause a sudden decrease in ruminal pH, which is associated with immunosuppression and inflammation (Kleen et al., 2003; Gozho et al., 2005). Under low ruminal pH conditions, increased amounts of free ruminal lipopolysaccharides (LPS) translocate into the blood, activating an inflammatory response (Gozho et al., 2005). While subacute ruminal acidosis (SARA) does not adversely affect calf performance during weaning transition, decreasing dietary calf starter consumption does not alleviate ruminal acidosis in calves (Laarman et al., 2012). In contrast, hay consumption might be important in mitigating ruminal acidosis in dairy calves during weaning transition (Laarman and Oba, 2011).

Ruminal bacteria can adapt adequately to dietary changes. For example, bacterial diversity and population size in the ruminal epithelium are affected by dietary changes (Liu et al., 2015), and epimural bacterial communities differ among cattle fed high-grain and forage diets (Petri et al., 2013a). In goats, a high-grain diet not only decreased ruminal pH but also caused a strong shift in the epimural bacterial community, which was associated with alterations in the relative expression of toll-like receptors in the ruminal epithelium (Liu et al., 2015). Fructose feeding increased the relative abundance of *Firmicutes* and decreased that of *Proteobacteria* after a short period, and *Streptococcus bovis* was specifically observed in fructose-fed heifers, which was identified by a false discovery rate analysis (Golder et al., 2014). Furthermore, rumen bacterial microbiota differed in bacterial diversity, richness, and composition between dairy cattle fed a control diet vs. a SARA-inducing diet (Mao et al., 2013). Among cattle with induced SARA, reductions in bacterial diversity and abundance of Gram-negative bacteria were observed, which were directly correlated with an increase in ruminal LPS levels (Mao et al., 2013).

Recent studies have found that rumen microbial communities are established soon after birth, before solid feed consumption (Jami et al., 2013; Rey et al., 2014). Rumen bacteria can be transmitted from the dam via the birth canal, teat surface, skin, or saliva; therefore, calf rumen bacterial communities depend greatly on maternal interactions (Chaucheyras-Durand and Ossa, 2014). Moreover, a diet of milk, compared with milk plus solid feed, fed to calves during the first 3 weeks of life differentially affected microbial communities in the gastrointestinal tract and feces, as well as body weight and ruminal pH and weight (Guzman et al., 2015). Some bacteria essential for mature rumen function can be detected as early as 1 day after birth, long before the ruminal bacterial community is established (Jami et al., 2013). *Prevotella* was observed to be the predominant genus upon a rapid increase in solid feed intake from 15 to 83 days of age (Rey et al., 2014) as well as in animals fed high-fiber diets (Jami et al., 2013). Although it has been demonstrated that rumen ecology is influenced by maternal dams, feeding materials, and rumen conditions from very soon after birth into adulthood, the correlation between ruminal pH and bacteria during weaning transition remains unclear.

The objective of this study was to investigate the relationship between ruminal pH and bacteria in calves fed calf starter with and without a forage diet during weaning transition. We hypothesized that forage consumption would mitigate adverse changes in ruminal pH and that bacteria would change due to these alterations.

## Materials and methods

### Animals and experimental design

All animals were cared for according to protocols approved by Iwate University Laboratory Animal Care and Use Committee. In total, 16 4-week-old Holstein bull calves were obtained from dairy farms and equipped with rumen cannulas by cannulation surgery 4 days after arrival. Calves were housed individually in 2.0 × 1.2-m pens with rubber mats and had free access to water throughout the study period. For the experiment, the calves were subjected to 3 weeks of adaptation (4–6 weeks of age), a preweaning phase (7 weeks of age), weaning transition (8 weeks of age), and a post-weaning phase (9–11 weeks of age). The diet was supplied in two equal portions at 08:00 and 16:30 daily, and daily total dry matter intake (DMI) was recorded individually for each calf throughout the study. The chemical compositions of the milk replacer, calf starter concentrate, and mixed forage (orchard and timothy hay) fed to calves are shown in Supplementary Table 1.

During the adaptation period, calves were fed 300 g/1.8 L commercial milk replacer (Meiji Feed Co., Kashima, Japan), 600 g calf starter (Meiji Feed Co., Kashima, Japan), and 200 g forage. At 6 weeks of age, calves (73.5 ± 4.2 kg; mean ± SE) were divided into two groups, the HAY (*n* = 8) and CON groups (*n* = 8). At 7 weeks of age, weaning was started in both groups by reducing the milk replacer to 150 g/0.9 L, and forage was restricted in the CON group until the end of the study. All calves were weaned at 8 weeks of age. From 7 to 11 weeks of age, the amount of calf starter was gradually increased from 800 to 1,200 g in the HAY group and 800 to 1,600 g in the CON group, while the amount of forage was concurrently increased from 200 to 400 g in the HAY group. Both groups had the same total DMI from weaning transition to a post-weaning phase. The amount of feeding was based on the Japanese Feeding Standard for Dairy Cattle.

### Sampling and measurements

Ruminal pH was measured continuously every 10 min throughout the experiment using a radio transmission system (YCOW-S; DKK-TOA Yamagata, Yamagata, Japan) as reported previously (Sato et al., 2012). The pH sensor was placed in the ventral sac of the rumen, and rumen fluid samples were collected from the ventral sac of the rumen, adjacent to the pH sensor, in the morning before feeding at 7, 8, 9, and 11 weeks of age (−1, 0, 1, and 3 weeks after weaning, respectively). Rumen fluid samples were immediately filtered through two layers of cheesecloth after sampling. For the VFA analysis, 2 mL 25% HO_3_P in 3N H_2_SO_4_ were added to 10 mL rumen fluid. Total VFA and VFA components (i.e., acetic acid, propionic acid, and butyric acid) were separated and quantified by gas chromatography (Model 135, Hitachi, Tokyo, Japan) using a packed glass column (Thermon-3000, 3%) on a Shimalite TPA 60–80 mesh support (Shinwa Chemical Industries Ltd., Kyoto, Japan). Filtered rumen fluid samples were stored at −80°C until further analysis.

### DNA isolation

For DNA isolation, rumen fluid samples were thawed, and 250 μL aliquots were centrifuged at 9,700 × g for 30 min, after which the supernatant was discarded. For each sample, the pellet was re-suspended in 300 μL TE buffer, and total bacterial DNA was extracted as described previously (Morita et al., 2007) with minor modifications. The mixture was incubated with 750 μg/mL lysozyme (Sigma-Aldrich, St. Louis, MO, USA) at 37°C for 90 min. Then, 10 μL purified achromopeptidase (Wako Pure Chemical Industries, Ltd., Osaka, Japan) were added at a concentration of 10,000 U/mL and incubated at 37°C for 30 min. The suspension was treated with 60 μL 1% sodium dodecyl sulfate and 1 mg/mL proteinase K (Merck Japan, Tokyo, Japan), and incubated at 55°C for 5 min. The lysate was treated with phenol/chloroform/isoamyl alcohol (Wako Pure Chemical Industries, Ltd.) and chloroform (Life Technologies Japan, Ltd., Tokyo, Japan). DNA was precipitated by adding 5M NaCl and 100% ethanol and centrifuged at 21,900 × g for 15 min. The DNA pellet was rinsed with 70% ethanol, dried, and dissolved in TE buffer. The purified DNA was quantified using a Biospec-nano (Shimadzu, Kyoto, Japan) and stored at −80°C until further analysis.

### DNA pyrosequencing

The V1/V2 region of the 16S rRNA gene was amplified using a forward primer (5′-CCA TCTCATCCCTGCGTGTCTCCGACTCAGNNNNNNNNNNAGRGTTTGATYMTGGCT CAG-3′) containing 454 primer A, a unique 10-bp barcode sequence for each sample (indicated as N), and 27Fmod (5′-AGR GTTTGATYMTGGCTCAG) in which the third base, A, in the original primer 27F was changed to R, as well as the reverse primer (5′-CCT ATCCCCTGTGTGCCTTGGCAGTCTCAGTGCTGCCTCCCGTAGGAG T-3′) containing 454 primer B and reverse primer 338R (5′-TGC TGCCTCCCGTAGGAGT). Amplified products of ~370 bp were confirmed using agarose gel electrophoresis, purified using AMPure XP magnetic purification beads (Beckman Coulter, Inc., Brea, CA, USA), and quantified using the Quant-iT PicoGreen dsDNA Assay Kit (Life Technologies Japan). Mixed samples were prepared by pooling approximately equal amounts of PCR amplicons from each sample and then subjected to 454 GS Junior (Roche Applied Science, Indianapolis, IN, USA) sequencing following the manufacturer's instructions. The sequencing data were deposited into the Sequence Read Archive (SRA) of NCBI1 and can be accessed via accession number SRP076881.

### Pyrosequencing data analysis

All pyrosequencing reads were filtered according to the procedure of Kim et al. (2013), who developed an analysis pipeline for barcoded 454 pyrosequencing of PCR amplicons in V1/V2, the region amplified by the 27Fmod/338R primers. Pyrosequencing reads were assigned to each sample based on the barcode sequence information. Resulting sequences that did not have PCR primer sequences at both sequence termini and those with an average quality value >25 were filtered out. Chimeras containing BLAST match lengths of >90% similarity with reference sequences in the database were removed. Finally, filter-passed reads were obtained for further analysis by trimming off both primer sequences. For the operational taxonomic unit (OTU) analysis, 16S reads were clustered using a 96% pairwise identity cutoff in the UCLUST program (www.drive5.com). Representative sequences for each OTU were assigned to bacterial species based on a BLAST search, with a 96% pairwise identity cutoff, against the 16S rRNA gene sequence database, constructed using Ribosomal Database Project tools (ver. 10.27; http://rdp.cme.msu.edu/), and against a reference genome database constructed from genome sequences collected from GenBank (ftp://ftp.ncbi.nih.gov/genbank/, November 2013).

A total of 506,717 filter-passed sequences were obtained from the analysis pipeline (Kim et al., 2013). Filter-passed reads were processed using MOTHUR (ver. 1.35, University of Michigan; http://www.mothur.org/wiki/; Schloss et al., 2009) and all samples were standardized by random subsampling to 3420 sequences per sample using the “sub.sample” command to generate rarefaction curves and calculate the abundance-based coverage estimator (ACE), Chao1 richness estimator, and Shannon diversity index, according to the Illumina MiSeq protocol described previously (Kozich et al., 2013). In order to obtain a non-redundant set of sequences, unique sequences were determined, and used to align against the SILVA reference alignment database (Pruesse et al., 2007); chimera were removed using chimera.uchime (http://drive5.com/uchime); sequences identified as being of eukaryotic origin were removed; the candidate sequences were screened and preclustered to eliminate outliers; and a distance matrix was generated from the resulting sequences. Sequences were clustered into OTU with a cutoff of 97% similarity. Rarefaction curve was generated at the level of 97% similarity level, which was calculated by the distance-based OTU (Schloss et al., 2011). For calculation of the non-parametric species richness estimators Chao 1 and ACE, the diversity index Shannon, the “summary.single” command was used. The unweighted UniFrac distance method (Lozupone and Knight, 2005) was used to perform a principal coordinates analysis (PCoA) with all OTU.

### Real-time quantitative PCR

Quantitative real-time PCR (qRT-PCR) was performed to evaluate the copy number of methyl-coenzyme M reductase α-subunit (*mcrA*) from total methanogens and 16S rRNA genes from *Fibrobacter succinogenes, Megasphaera elsdenii, Ruminococcus albus, Ruminococcus flavefaciens, Streptococcus bovis*, and *Selenomonas ruminantium* using SYBR green (iQ SYBR Green Supermix, Bio-Rad, Hercules, CA, USA) with the MiniOpticon Real-Time PCR system (Bio-Rad). Primer pairs (Supplementary Table 2) were selected to detect bacterial species closely associated with dietary changes and other bacterial species. Each sample contained 10 ng DNA, 2 × SYBR green, and 0.6 μM each primer in a final volume of 20 μL. Amplification conditions were as follows: 95°C for 3 min, 40 cycles of 10 s at 95°C, 20 s at 63°C (for total methanogens), 60°C (for *F. succinogenes*), 58°C (for *M. elsdenii*), 57°C (for *S. bovis* and *S. ruminantium*), or 55°C (for *R. albus* and *R. flavefaciens*), and 30 s at 72°C. The fluorescence signal was collected at the end of each cycle. To obtain melting curve data, the temperature was increased in 0.5°C increments from 65 to 94°C. A standard curve for each primer pair was constructed from recombinant plasmid DNA containing 16S rRNA inserts of DNA purified from a pure culture of the target species. The strains used for plasmid preparation were as followed: *Methanobrevibacter ruminantium* JCM13430 (DSM1093), *F. succinogenes* ATCC19169, *M. elsdenii* ATCC25940, *R. albus* ATCC27210, *R. flavefaciens* ATCC19208, *S. bovis* ATCC33317, and *S. ruminantium* ATCC12561. Plasmid DNA was quantified and subjected to seven sequential 10-fold dilutions. Data were collected and processed using CFX Manager software ver. 1.5 (Bio-Rad).

### Statistical analysis

Ruminal pH data were summarized as 24- and 1-h means. VFA, relative abundance of bacterial phyla and genera, ruminal bacterial diversity indices, and bacterial species copy numbers were summarized at −1, 0, 1, and 3 weeks after weaning. All numerical data were expressed as means ± standard error (SE) and analyzed using Prism ver. 7.01 (GraphPad Software, Inc., La Jolla, CA, USA). The normality of the distribution of variables was tested using Shapiro-Wilk test, and non-normal data were root-square transformed before analysis. Total DMI at 6 weeks of age, 24- and 1-h mean ruminal pH, total VFA concentration, proportions of individual VFA, relative abundance of each OTU, ruminal bacterial diversity indices, and bacterial species copy number were compared between the HAY and CON groups using two-way repeated measures ANOVA, and multiple testing false discovery rate (FDR) *p*-value was determined (Benjamini and Hochberg, 1995). The statistical model included the main effects of diet, time, and their interaction, plus the random effect of animal. Differences were considered to be significant at *P* < 0.05, and trends suggesting possible significance were determined at *P* < 0.10.

## Results

### Daily total DMI, ruminal pH, and VFAs

**Figure 1:**
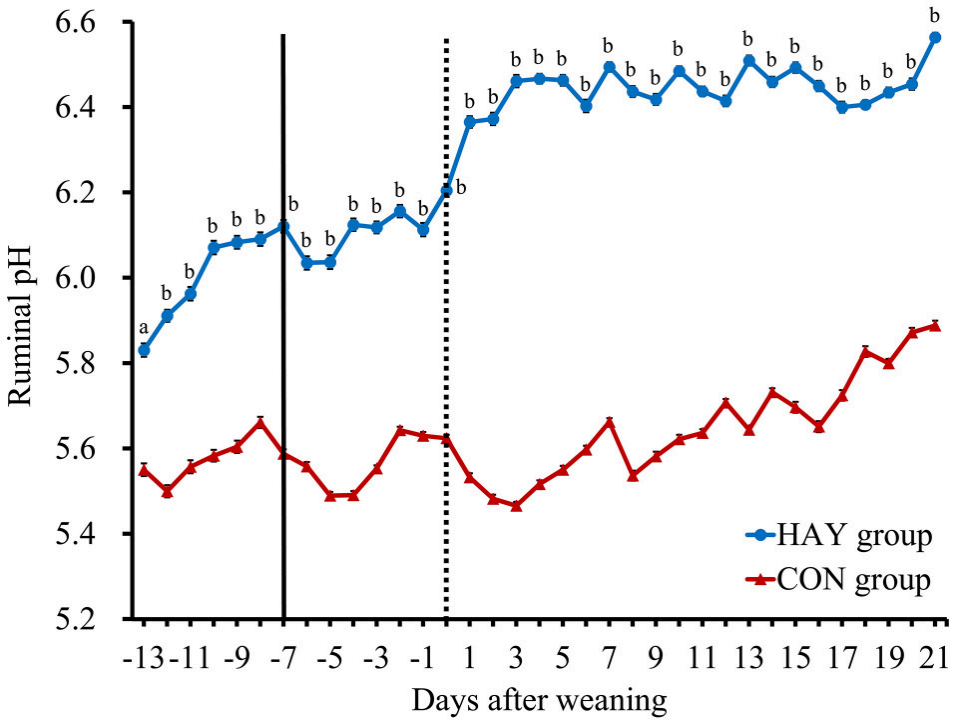
**Daily changes in the 24-h mean ruminal pH in Holstein bull calves fed calf starter with (HAY group, *n* = 8) and without (CON group, *n* = 8) forage**. Values represent the means ± SE. The dotted and solid lines represent the start of the experiment and time of weaning, respectively. ^a^ and ^b^ denote significant differences (*P* < 0.05 and *P* < 0.01, respectively) between the HAY and CON groups within the same day.

**Figure 2:**
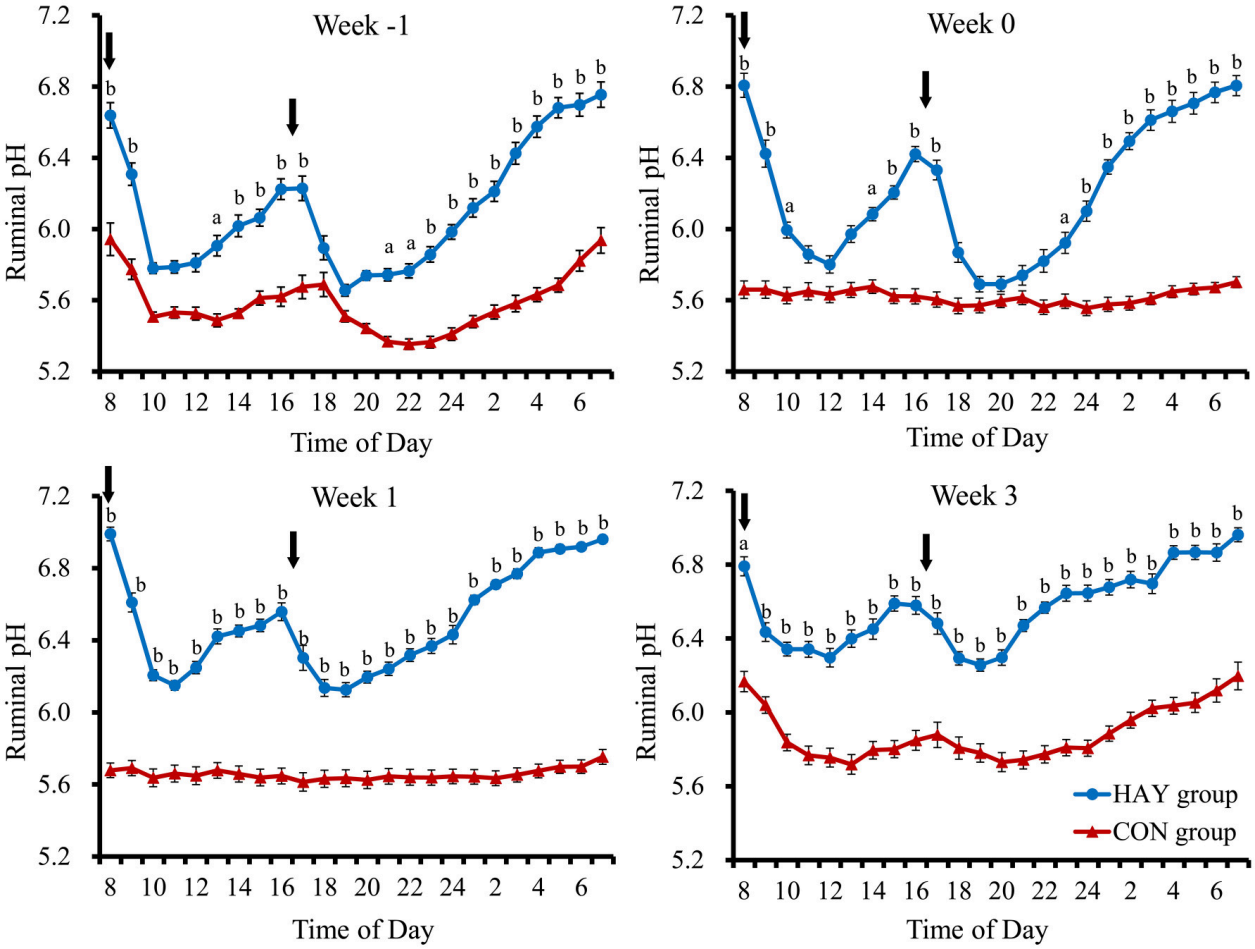
**Diurnal changes in the 1-h mean ruminal pH in Holstein bull calves fed calf starter with (HAY group, *n* = 8) and without (CON group, *n* = 8) forage**. Week −1, Week 0, Week 1, and Week 3 represent calves at − 1, 0, 1, and 3 weeks after weaning, respectively. Values represent the means ± SE. Arrows indicate feeding times (08:00 and 16:30). ^a^ and ^b^ denote significant differences (*P* < 0.05 and *P* < 0.01, respectively) between the HAY and CON groups at the same time point.

No significant difference (*P* = 0.283) in total DMI was observed at 6 weeks of age between the two groups. The 24-h mean ruminal pH was significantly (*P* < 0.01) different between the HAY and CON groups (Figure 1). The interaction of diet × time of sampling and the effect of time were significant (*P* < 0.01) for the 24-h mean ruminal pH between the groups. The ruminal pH in both groups decreased after the morning feeding and then began to increase up to 3 h after feeding at −1 week after weaning (Figure 2). Diurnal changes in the 1-h mean ruminal pH were observed at −1, 0, 1, and 3 weeks after weaning in the HAY group (Figure 2). However, they were not observed at 0 and 1 weeks in the CON group and were weak at 3 weeks after weaning. The interaction of diet × time of sampling and the effect of time were significant (*P* < 0.01) for the 1-h mean ruminal pH between the two groups throughout the experiment.

Significant differences (*P* < 0.01) were observed in the proportions of acetate and propionate between the HAY and CON groups (Table 1). The proportion of acetate was significantly (*P* < 0.05) higher at 1 and 3 weeks in the HAY group, and the proportion of propionate was significantly (*P* < 0.05) higher at 1 and 3 weeks after weaning in CON group. The proportion of butyrate was significantly (*P* < 0.05) higher at 3 weeks after weaning in the HAY group. Others that VFA components exclude acetate, propionate, and butyrate from total VFA was significantly (*P* < 0.05) higher at 0 week after weaning in the HAY group. In addition, a significant (*P* < 0.05) difference in the ruminal acetate-to-propionate ratio was observed between the two groups. Furthermore, the ruminal acetate-to-propionate ratios were significantly (*P* < 0.05) lower at 1 and 3 weeks after weaning in the CON group. The effect of time was not significant for the ruminal acetate-to-propionate ratio, total VFA concentration, and individual VFA proportions. The interaction of diet × time of sampling was significant for the proportion of acetate, propionate, butyrate, and the ruminal acetate-to-propionate ratio, whereas others VFA component was differed slightly (*P* = 0.065) between the HAY and CON groups.

**Table 1:** **Total VFA concentration and individual VFA proportions of rumen fluid in Holstein bull calves fed calf starter with (HAY group) and without (CON group) forage**.

**Item**	**HAY group (*n* = 8)**				**CON group (*n* = 8)**					***P*****-value**		
	**Week −1**	**Week 0**	**Week 1**	**Week 3**	**Week −1**	**Week 0**	**Week 1**	**Week 3**	**SEM**	**Time (T)**	**Diet (D)**	**T × D**
Total VFA, m*M*	75.62	83.09	92.44	103.68	98.35	85.54	105.20	104.40	5.16	0.115	0.599	0.609
**VFA, mol/100 mol**												
Acetate	57.39^a^	56.12^a^	63.47^a^	59.93^a^	56.05^a^	53.58^a^	49.13^b^	50.02^b^	0.86	0.471	0.005	<0.001
Propionate	29.43^a^	29.43^a^	22.71^a^	24.25^a^	30.01^a^	35.07^a^	38.10^b^	37.24^b^	1.04	0.520	0.006	<0.001
Butyrate	8.43^a^	8.79^a^	8.87^a^	10.39^a^	9.76^a^	7.30^a^	7.46^a^	7.83^b^	0.32	0.261	0.279	0.050
Others^1^	4.74^a^	5.66^a^	4.96^a^	5.43^a^	4.18^a^	4.05^b^	5.32^a^	4.92^a^	0.16	0.175	0.192	0.065
Acetate: propionate	2.08^a^	2.08^a^	2.93^a^	2.65^a^	2.12^a^	1.60^a^	1.33^b^	1.45^b^	0.11	0.395	0.015	<0.001

1 *VFA components excluded acetate, propionate, and butyrate from total VFA*.

a, b *Means within a row, different superscripts differ (P < 0.05) between the HAY and CON groups at the same week point*.

### Bacterial diversity analysis

**Figure 3:**
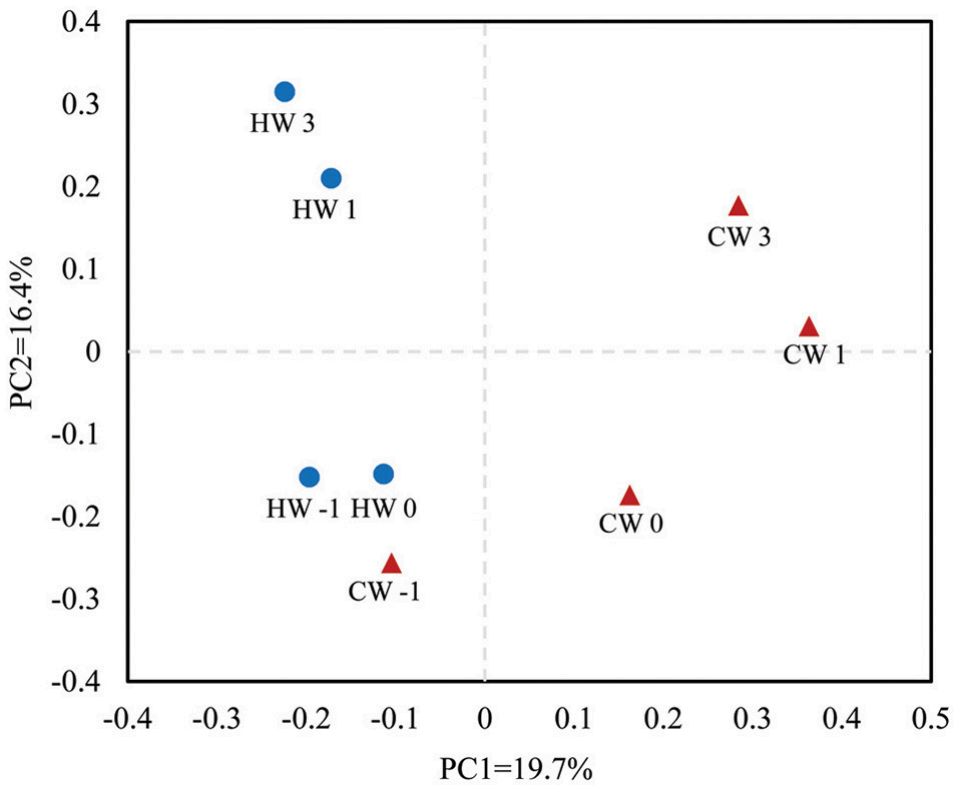
**Principal coordinate analysis plots generated from the 454 pyrosequencing data**. PC1 and PC2 represent principal components 1 and 2, respectively. HW-1, HW0, HW1, and HW3 represent calves at −1, 0, 1, and 3 weeks, respectively, after weaning in the HAY group (*n* = 8), and CW-1, CW0, CW1, and CW3 represent calves at −1, 0, 1, and 3 weeks, respectively, after weaning in the CON group (*n* = 8). Blue circles represent the HAY group and red triangles the CON group.

The rarefaction curve of ruminal bacterial microbiota calculated at a 97% similarity level indicated that the HAY group had higher bacterial diversity than that of the CON group (Supplementary Figure S1). An unweighted UniFrac distance analysis in MOTHUR was used to evaluate β-diversity across the samples. The PCoA results indicated that the HAY group was distinctly separate from the CON group in the plot from weaning transition to post-weaning phase (Figure 3; PC1 + PC2 = 36.1%). The HAY and CON groups of the plot displayed close distance at −1 week, whereas those of the two groups were separated by age from 0 to 3 weeks after weaning. The plots of each group showed marked similarities between 1 and 3 weeks after weaning.

Bacterial diversity was estimated in the HAY and CON groups each week using OTUs, ACE, Chao1, and the Shannon index (Table 2). The OTUs, ACE, and Chao1 results differed significantly (*P* < 0.05) between the two groups and decreased after −1 week after weaning. However, they rebounded at 0 and 1 weeks after weaning in the HAY and CON group, respectively. The Shannon index analysis showed a tendency (*P* = 0.055) toward different bacterial diversity between the two groups. The interaction of diet × time of sampling and the effect of time were not significant for the OTUs, ACE, Chao1, and Shannon index between the HAY and CON groups.

**Table 2:** **Ruminal bacterial diversity calculated from 454 pyrosequencing data at a 97% similarity level in Holstein bull calves fed calf starter with (HAY group) and without (CON group) forage**.

**Item**	**HAY group (*n* = 8)**				**CON group (*n* = 8)**					***P*****-value**		
	**Week −1**	**Week 0**	**Week 1**	**Week 3**	**Week −1**	**Week 0**	**Week 1**	**Week 3**	**SEM**	**Time (T)**	**Diet (D)**	**T × D**
OTUs^a^	272	243	271	284	222	205	186	192	9	0.612	0.013	0.426
Chao1	489	426	477	515	388	368	338	366	16	0.490	0.012	0.538
ACE^b^	669	563	597	675	500	482	420	477	21	0.330	0.007	0.647
Shannon index	3.83	3.67	3.58	3.81	3.27	3.18	3.33	3.28	0.07	0.795	0.055	0.695

a *Operational taxonomic units*.

b *Abundance-based coverage estimator*.

### Bacterial abundance

**Figure 4:**
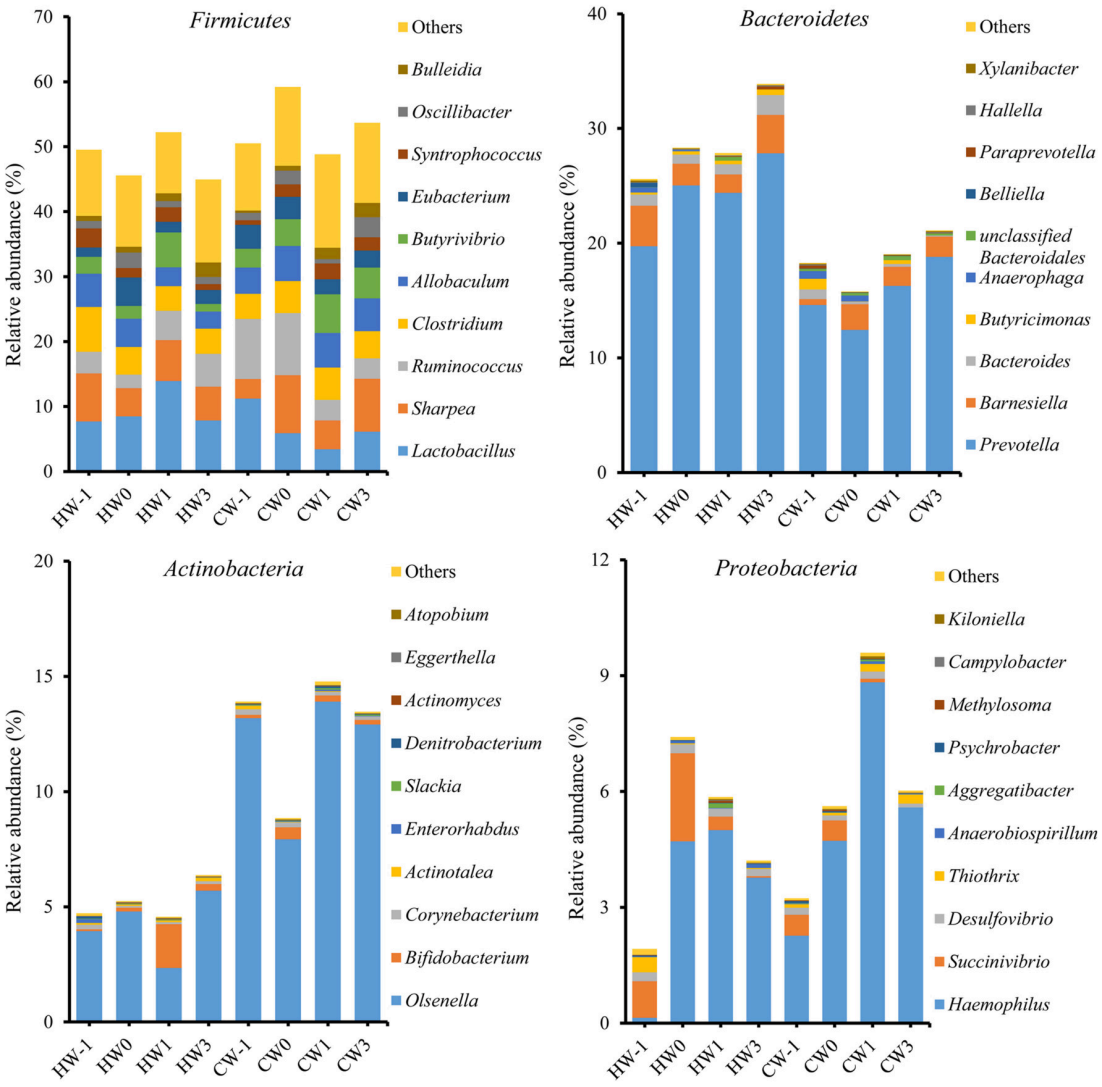
**Relative abundances of the four major bacterial phyla and genus profiles identified by 454 pyrosequencing**. Data are shown as the percentages of the total identified sequences per group. HW-1, HW0, HW1, and HW3 represent calves at −1, 0, 1, and 3 weeks, respectively, after weaning in the HAY group (*n* = 8), and CW-1, CW0, CW1, and CW3 represent calves at −1, 0, 1, and 3 weeks, respectively, after weaning in the CON group (*n* = 8).

A total of 16 bacterial phyla and 1 candidate phylum were identified within the ruminal bacteria. Of the major phyla, *Firmicutes, Bacteroidetes, Actinobacteria*, and *Proteobacteria* were the most abundant in both groups, accounting for 88.56% of the total ruminal bacteria (Figure 4). The remaining phyla had low relative abundances of < 1%, reporting threshold of the relative abundance. A total of 341 bacterial genera were identified, and the relative abundances of 327 genera comprised < 1% of the total sequences. There were 72 bacterial genera specific to the HAY group, 47 bacterial genera specific to the CON group, and 222 bacterial genera common to both groups. *Prevotella* was the predominant genus in both groups. *Prevotella* (22.84%), *Lactobacillus* (9.48%), and *Ruminococcus* (5.41%) were the most abundant bacterial genera in the HAY group, while *Prevotella* (18.16%), *Olsenella* (10.50%), and *Lactobacillus* (7.38%) were the most abundant genera in the CON group. Ruminal bacterial phylum and genus which had the relative abundance of below our reporting threshold (< 1%) were excluded from the report.

The relative abundances of bacterial phyla (*P* < 0.05), including *Bacteroidetes, Actinobacteria*, and *Tenericutes*, and the *Firmicutes*-to-*Bacteroidetes* ratio (*P* < 0.01) differed significantly between the HAY and CON groups (Table 3). The genus *Prevotella*, which belongs to *Bacteroidetes*, was the only bacterial genus that differed significantly (*P* < 0.05) between the two groups, and there were no significant differences in other bacterial genera. The diet × time of sampling interaction was significant for *Dialister* (*P* < 0.01) and unclassified *Lachnospiraceae* (*P* < 0.05) and showed a tendency toward significance (*P* = 0.072) for *Syntrophococcus*. The effect of time was significant for *Bulleidia* (*P* < 0.05) between the two groups.

**Table 3:** **Relative abundances (% of total sequences) of the major bacterial phyla and genera identified by 454 pyrosequencing in Holstein bull calves fed calf starter with (HAY group) and without (CON group) forage**.

**Item**	**HAY group (*n* = 8)**				**CON group (*n* = 8)**					***P*****-value**		
	**Week −1**	**Week 0**	**Week 1**	**Week 3**	**Week −1**	**Week 0**	**Week 1**	**Week 3**	**SEM**	**Time (T)**	**Diet (D)**	**T × D**
**BACTERIAL PHYLUM**												
*Firmicutes*	49.53	45.56	52.23	44.93	50.51	59.19	48.81	53.68	1.52	0.903	0.123	0.199
*Bacteroidetes*	25.60	28.33	27.85	33.90	18.28	15.79	19.03	21.12	1.40	0.203	0.013	0.727
*Actinobacteria*	4.72	5.25	4.56	6.38	13.91	8.85	14.78	13.46	1.51	0.660	0.002	0.845
*Proteobacteria*	1.92	7.41	5.86	4.21	3.23	5.62	9.59	6.03	0.97	0.176	0.637	0.709
*Firmicutes*:*Bacteroidetes*	2.09	1.84	1.96	1.43	11.06	7.56	4.20	6.50	1.10	0.736	0.022	0.777
**BACTERIAL GENUS**												
*Prevotella*	19.74	25.05	24.39	27.84	14.63	12.44	16.27	18.80	1.41	0.226	0.039	0.669
*Lactobacillus*	7.68	8.50	13.93	7.84	11.25	5.91	3.44	6.13	1.31	0.269	0.889	0.437
*Olsenella*	3.94	4.80	2.35	5.71	13.19	7.94	13.91	12.91	1.51	0.763	0.131	0.507
*Ruminococcus*	3.34	2.07	4.50	5.05	9.26	9.57	3.17	3.12	0.91	0.805	0.127	0.120
*Sharpea*	7.41	4.33	6.30	5.22	2.99	8.91	4.42	8.16	1.00	0.886	0.916	0.237
*Clostridium*	6.89	4.28	3.80	3.91	3.86	4.93	4.95	4.19	0.54	0.747	0.919	0.427
*Haemophilus*	0.14	4.71	5.00	3.77	2.27	4.73	8.83	5.59	0.97	0.137	0.461	0.887
*Allobaculum*	5.14	4.34	2.92	2.61	4.05	5.41	5.36	5.05	0.77	0.890	0.657	0.576
*Eubacterium*	1.43	4.37	1.62	2.16	3.68	3.46	2.34	2.60	0.35	0.167	0.476	0.381
*Butyrivibrio*	2.60	1.97	5.36	1.15	2.92	4.08	5.92	4.76	0.80	0.273	0.529	0.740
*Barnesiella*	3.54	1.88	1.62	3.34	0.51	2.24	1.67	1.77	0.45	0.842	0.428	0.366
*Oscillibacter*	1.14	2.39	0.92	1.08	1.13	2.10	0.67	3.11	0.46	0.132	0.422	0.575
*Syntrophococcus*	2.95^a^	1.45^a^	2.27^a^	0.92^a^	0.68^b^	1.93^a^	2.41^a^	2.05^a^	0.27	0.615	0.856	0.072
Unclassified *Clostridiales*	0.32	1.36	0.78	0.87	0.99	0.81	2.51	1.04	0.20	0.184	0.128	0.323
*Bulleidia*	0.75	0.89	1.20	2.23	0.38	0.75	1.77	2.17	0.21	0.014	0.998	0.828
*Roseburia*	0.62	1.09	0.45	0.79	0.36	2.81	0.46	1.55	0.36	0.254	0.602	0.645
*Dialister*	1.07^a^	0.38^a^	0.63^a^	0.51^a^	0.43^a^	0.71^a^	1.38^a^	1.90^b^	0.16	0.112	0.380	0.007
Unclassified *Lachnospiraceae*	0.50^a^	0.94^a^	0.47^a^	1.62^a^	0.93^a^	0.52^a^	2.31^b^	0.40^a^	0.21	0.510	0.784	0.029

a, b *Means within a row, different superscripts differ (P < 0.05) between the HAY and CON groups at the same week point*.

### Copy number of bacterial 16S rRNA

The copy numbers of *R. albus* differed significantly (*P* < 0.01), while those of *R. flavefaciens* differed slightly (*P* = 0.087) between the HAY and CON groups (Table 4). The higher copy numbers of *R. flavefaciens, R. albus*, and *S. bovis* in the HAY group were observed compared with the CON group. Total methanogens (*P* < 0.05) and *M. elsdenii* (*P* < 0.01) copy numbers were affected significantly by the effect of time. The diet × time of sampling interaction was not significant for changes in the bacterial species copy numbers.

**Table 4:** **Copy number of 16S rRNA genes identified from qRT-PCR in Holstein bull calves fed calf starter with (HAY group) and without (CON group) forage**.

**Item**	**HAY group (*n* = 8)**				**CON group (*n* = 8)**					***P*****-value**		
	**Week −1**	**Week 0**	**Week 1**	**Week 3**	**Week −1**	**Week 0**	**Week 1**	**Week 3**	**SEM**	**Time (T)**	**Diet (D)**	**T × D**
Total methanogens	3.46	3.08	2.66	2.52	3.35	3.29	2.65	2.49	0.12	0.034	0.987	0.881
*Fibrobacter succinogenes*	3.72	4.05	4.07	4.29	3.69	2.97	3.24	3.60	0.20	0.307	0.159	0.260
*Megasphaera elsdenii*	4.57	4.77	4.94	4.83	4.45	4.30	4.75	4.70	0.18	0.007	0.380	0.376
*Ruminococcus albus*	4.33	4.63	4.52	4.95	3.95	3.66	3.81	4.10	0.17	0.173	0.010	0.295
*Ruminococcus flavefaciens*	4.92	4.86	5.26	5.09	4.81	4.33	4.41	4.82	0.17	0.308	0.087	0.166
*Streptococcus bovis*	2.07	2.35	2.41	2.81	1.86	1.92	1.89	2.06	0.12	0.327	0.166	0.473
*Selenomonas ruminantium*	5.53	5.61	5.49	5.41	5.21	5.46	5.52	5.66	0.18	0.602	0.933	0.140

*Values are expressed as log*.

## Discussion

This study aimed to identify the long-term relationship between ruminal pH and bacteria during weaning transition, and rumen fluid samples were collected in the morning before feeding to minimize the short-term effects of diet on ruminal bacteria that observed in grain-, fructose-, and histidine-fed dairy heifers (Golder et al., 2014). Total DMI at 2 weeks before weaning and the analyses of total VFA, individual proportion of VFA, PCoA, bacterial diversity indices (OTUs, Chao1, and ACE), and the copy number of ruminal bacteria at 1 week before weaning indicate that there was no difference in DMI and the rumen environment between the two groups. Moreover, ruminal bacteria was affected by low ruminal pH regardless of sample type, such as rumen fluid, rumen contents, and rumen epithelium (Mao et al., 2013; Liu et al., 2015; Sato, 2016). In our study, the feeding of calf starter with forage alleviates the depression of 24-h mean ruminal pH compared with the feeding of only calf starter, and the lower ruminal pH in the CON group was associated with greater consumption of calf starter. The diurnal changes in the 1-h mean ruminal pH observed in the HAY group play an important role in increasing the 24-h mean ruminal pH. However, the relationship between ruminal pH regulation and VFA absorption could not be explained in this study due to insufficient sampling throughout the day. Production of acetate, butyrate, and propionate and ruminal acetate-to-propionate ratios in the CON group were consistent with general feature of starch source feeding in calves (Laarman et al., 2012; Castells et al., 2013).

Khan et al. (2011) found that providing chopped hay to calves fed a high volume of milk at an early age improved their total solid feed intake and was beneficial for rumen development. Moreover, calves fed diets supplemented with oat hay had increased ruminal pH than that of calves offered no forage (Castells et al., 2013), and providing chopped hay was necessary soon after weaning to improve calf performance (Terré et al., 2013). In our study, calves in both groups had a ruminal pH of < 5.8 at −1 week after weaning (Figure 2), which is applied as a diagnostic of SARA in dairy calves (Laarman et al., 2012). However, the ruminal pH of < 5.8 was mitigated in calves fed calf starter with forage, while that of calves fed only calf starter was maintained throughout the experiment. The higher ruminal pH in the HAY group compared with the CON group could have been caused by hay intake, stimulating chewing and salivary buffer flow (Laarman et al., 2012), and by part of the concentrate, the fermentation source, being replaced by forage during weaning transition.

The impact of dietary changes on rumen microbial composition has been investigated in several ruminants using a variety of molecular techniques. For example, terminal restriction fragment length polymorphism analysis indicated that the predominant rumen bacterial shift during SARA was a decline in the Gram-negative *Bacteroidetes*, induced by either grain or alfalfa pellets (Khafipour et al., 2009). In addition, 454 pyrosequencing analysis showed that the relative abundances of the phyla *Bacteroidetes* and *Proteobacteria* were reduced by consumption of concentrated feed in cattle with repeatedly induced SARA (Sato, 2016). In our study, lower relative abundances of the phyla *Bacteroidetes* and *Tenericutes* and Gram-negative bacteria in the CON group could be partly explained by a low rumen pH, which can lead to death and lysis of Gram-negative bacteria (Nagaraja and Titgemeyer, 2007). The higher *Firmicutes*-to-*Bacteroidetes* ratio in the CON group, due to a lower relative abundance of *Bacteroidetes*, was consistent with a previous study (Golder et al., 2014). In both groups, *Firmicutes* was the most relatively abundant Gram-positive bacteria, suggesting an increase in bacterial species that were metabolically capable of consuming newly available fermentable carbohydrates (Mao et al., 2013).

Liu et al. (2015) reported that a high-grain diet decreased the ruminal pH and resulted in lower bacterial diversity of the rumen epithelial community than hay diet. In this study, the ruminal bacterial diversity was also affected by diet, and feeding calf starter decreased 24-h mean ruminal pH and modified the composition of the ruminal bacterial microbiota at a 97% similarity level. Late increase in OTUs, ACE, and Chao1 observed in the CON group indicated that feeding only calf starter may signify the decline in rumen bacterial diversity during weaning transition. Furthermore, the higher ruminal bacterial diversity indices identified in the HAY group at a post-weaning phase suggested that feeding calf starter with forage enhances the increase in rumen bacterial diversity after weaning. Therefore, dietary forage supplementation that increases 24- and 1-h mean ruminal pH would increase rumen bacterial diversity and promote rapidly recovery from damage during weaning transition.

The most common genera of bacteria detected in the rumen of cattle linked to SARA and acidic challenge are *Lactobacillus* and *Streptococcus* (Petri et al., 2013b). Moreover, the number of *Lactobacillus* and *S. bovis* in the rumen contents of dairy cows during the transition period increased by switching from a low- to a high-grain diet (Wang et al., 2012). In our study, the most abundant lactate-producing genera were the relative abundance of *Lactobacillus* (9.48%) and *Olsenella* (10.50%) in the HAY and CON groups, respectively. The growth rate of *Lactobacillus* decreased linearly with increases in the concentration of sugars, mostly due to the osmotic stress exerted by the sugars (Narendranath and Power, 2005). Moreover, *Olsenella, Atopobium*, and *Bifidobacterium*, which constituted the lactic acid-producing bacteria *sensu lato* (Inês et al., 2008), accounted for up to 97.5% of the total relative abundance of *Actinobacteria* genera in the CON group. Therefore, it can be assumed that feeding relatively higher amount of starch source in the CON group affects the composition of lactate-producing bacteria and their growth might be enhanced by increasing the amount of calf starter feed in both groups.

*Streptococcus* and *S. bovis* were not affected by diet in this study. The relative abundance of *Streptococcus* identified by 454 pyrosequencing was below our reporting threshold, and the qRT-PCR results showed that *S. bovis* copy numbers did not differ significantly between the two groups, although they were higher in the HAY group at 3 weeks after weaning compared with the CON group. Because *S. bovis* is not always the main cause of rumen acidity (Hungate, 1966), not all studies have observed increases in or even identified *S. bovis* in grain-fed cattle (Tajima et al., 2000; Klieve et al., 2003). *Ruminococcus* bacteria, known as cellulolytic bacteria, was higher in the CON group at a preweaning phase and weaning transition in the 454 pyrosequencing results, while the qRT-PCR analysis revealed the higher *R. albus* and *R. flavefaciens* copy numbers in the HAY group at a post-weaning phase. Because lower amounts of *Ruminococcus* were likely due to the decrease in forage supplementation and not specifically a result of acidosis (Petri et al., 2013b), the higher *R. albus* and *R. flavefaciens* copy numbers in the HAY group at a post-weaning phase might have been induced by forage supplementation in this study. Although *Ruminococcus* consists largely of cellulolytic bacteria, there are also *Ruminococcus* species, such as *Rumincoccus bromii*, that can utilize starch (Klieve et al., 2007). Therefore, a major inconsistency was observed in the analysis results between the two methods for *Ruminococcus* due to the increase in starch fermentable *Ruminococcus* during weaning transition.

Lactate-metabolizing species such as *M. elsdenii* increase proportionately as the bacterial community adapts to more readily fermentable carbohydrates (Huber, 1976), which also leads to an increase in the prevalence of starch-fermenting bacteria such as *S. bovis* (Hungate, 1966). Ruminal methanogens contribute to eliminating reducing equivalents produced by carbohydrate-fermenting bacteria and protozoa by removing hydrogen generated during fermentation (Whitman et al., 2006). Aschenbach et al. (2011) reported that *M. elsdenii* populations were synchronized with *S. bovis* populations, indicating that they could assist in preventing lactic acid acidosis (Russell et al., 1981). However, according to our qRT-PCR analysis, the *M. elsdenii* copy number was changed with a low relative abundance of *Streptococcus* and *S. bovis* copy number in both groups, indicating that *M. elsdenii* might be associated with relatively more associated with *Lactobacillus* in the HAY group and *Olsenella* in the CON group than other lactate-producing species. Notably, increasing the amount of total DMI could increase the rate of passage from the rumen, thereby leaving less time for microbial fermentation (Huhtanen and Kukkonen, 1995). Increased passage rates could shift methanogenesis to the hindgut and manure (Hindrichsen et al., 2006), which could explain the gradual reduction in ruminal total methanogens observed in both groups, concurrent with the gradual increase in total DMI during weaning transition.

Due to their capacity to use a large variety of substrates, including starches, other non-cellulosic polysaccharides, and simple sugars, as energy sources to produce succinate as the major fermentation end product (Purushe et al., 2010), *Prevotella* bacteria can dominate and thrive under a range of diets (Stevenson and Weimer, 2007; Bekele et al., 2010). Moreover, *Prevotella ruminicola* was found in 1-day-old calves, and increased in number by day 3 (Jami et al., 2013). Although most *Prevotella* strains in the rumen represent species other than the classical ruminal *Prevotella* spp., recent studies have observed a clear predominance of *Prevotella* in bacterial populations. For example, the relative abundance of this genus accounted for up to 19.7% of total bacteria, whereas the representative species *Prevotella bryantii* and *P. ruminicola* accounted for only 0.6 and 3.8%, respectively (Bekele et al., 2010). Rey et al. (2014) suggested that *Prevotella* is associated with diets containing solid food; calves that start eating solid foods earlier tended to develop rumen bacterial communities similar to those of adults earlier. In our study, *Prevotella* had the greatest relative abundance in the both groups, and the positive correlation between the relative abundance of *Prevotella* and solid feed consumption was consistent with previous research (Rey et al., 2014). These results indicate that *Prevotella* could constitute one of the most crucial members of the ruminal bacteria in dairy calves during weaning transition.

To our knowledge, little is known regarding how and when ruminal bacteria establish a stable rumen microbiome in dairy calves. In humans, at ~1–2 years of age, the infant gut microbiome undergoes its second shift, and a stable adult microbiome begins to emerge, consistent with the establishment of a varied solid food diet (Bergström et al., 2014). In ruminant studies, changes in the rumen bacterial community caused by a transition from liquid to solid feed consumption have been mostly consistent with human studies (Jami et al., 2013; Rey et al., 2014). Therefore, the microbial shifts caused by dietary changes during weaning transition in this study were closely associated with the establishment of a mature rumen microbiome. However, dietary factors such as the composition of calf starter and forage type and timing of introduction should be considered carefully, although the causes and effects of the relationship between these factors have not been established (Yáñez-Ruiz et al., 2015). While this study contributes to a greater understanding of the response of the ruminal bacteria to dietary factors, further studies are needed to clarify the effects of diet on the establishment of ruminal bacteria from immature to mature animals.

## Conclusions

We investigated the relationship between ruminal pH and bacteria in calves fed calf starter with and without forage during weaning transition. The results supported our hypothesis that feeding calf starter with and without forage differentially affected the rumen environment. Feeding calf starter with forage mitigates the depression of 24-h mean ruminal pH due to diurnal changes in ruminal pH, in particular, a rebound from a rapid decrease in ruminal pH after feeding. Bacterial diversity was greater and recovered more rapidly from damage during weaning transition in the HAY group. Changes in the relative abundance and copy number observed in phyla, genus, and species might have affected the establishment of fermentative ruminal functions during weaning transition. This study increased understanding of the response of ruminal pH and bacteria to dietary factors in calves.

## Author contributions

YK carried out majority of the experiment including animal care, DNA isolation, real-time PCR, and pyrosequencing data analysis and interpretation; TI, RN, and NO were responsible for animal care, VFA analysis and DNA isolation; KI, and SS contributed to the conception of the project; The manuscript was prepared by YK and SS.

### Conflict of interest statement

The authors declare that the research was conducted in the absence of any commercial or financial relationships that could be construed as a potential conflict of interest.

## Supplementary material

Supplementary Figure S1 **Rarefaction curves generated from the 454 pyrosequencing data**. HW-1, HW0, HW1, and HW3 represent calves at −1, 0, 1, and 3 weeks, respectively, after weaning in the HAY group (*n* = 8), and CW-1, CW0, CW1, and CW3 represent calves at −1, 0, 1, and 3 weeks, respectively, after weaning in the CON group (*n* = 8).

Supplementary Table S1 **Chemical composition of milk replacer, calf starter concentrate, and mixed hay fed to calves**.

Supplementary Table S2 **Primer sequences used for qRT-PCR**.
