# Supplementary figures and images for: Effects of Dietary Forage and Calf Starter Diet on Ruminal pH and Bacteria in Holstein Calves during Weaning Transition
Source: Front Microbiol. 2016 Oct 21;7:1575. doi: 10.3389/fmicb.2016.01575 (PMC5073099; doi:10.3389/fmicb.2016.01575)

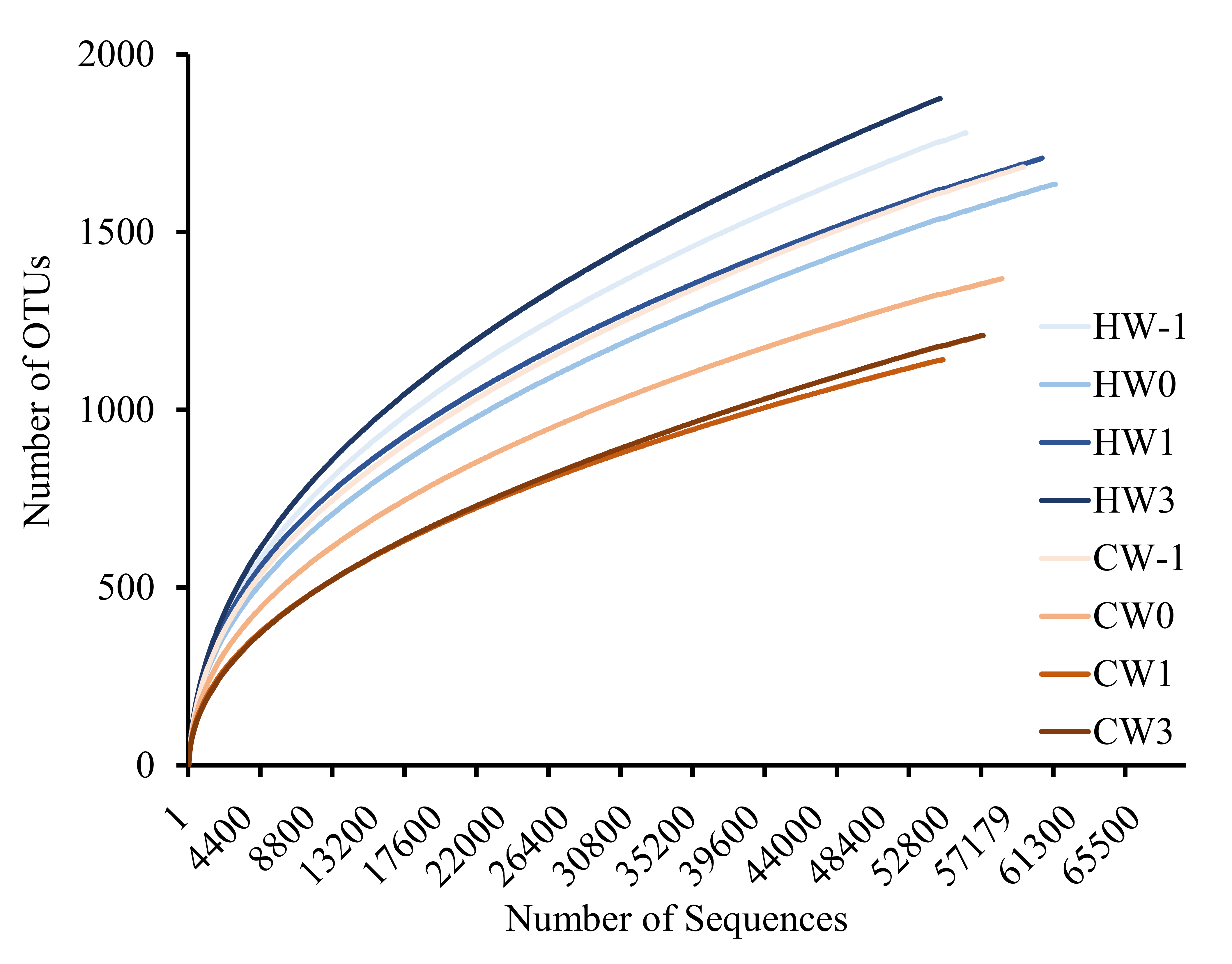

Supplement: Supplementary Figure S1 — Rarefaction curves generated from the 454 pyrosequencing data. HW-1, HW0, HW1, and HW3 represent calves at −1, 0, 1, and 3 weeks, respectively, after weaning in the HAY group (n = 8), and CW-1, CW0, CW1, and CW3 represent calves at −1, 0, 1, and 3 weeks, respectively, after weaning in the CON group (n = 8). [file Image1.jpg]
