# Supplementary material for: Effects of Dietary Forage and Calf Starter Diet on Ruminal pH and Bacteria in Holstein Calves during Weaning Transition
Source: Front Microbiol. 2016 Oct 21;7:1575. doi: 10.3389/fmicb.2016.01575 (PMC5073099; doi:10.3389/fmicb.2016.01575)
Supplement: Supplementary Table S1 — Chemical composition of milk replacer, calf starter concentrate, and mixed hay fed to calves. [file Table1.DOCX]

**Supplementary Table S1. Chemical composition of milk replacer, calf starter concentrate, and mixed hay fed to calves**

| Compositon | Milk replacer | Calf starter concentrate | Mixed hay^1^ |
| --- | --- | --- | --- |
| DM (%) | 96.9 | 88.1 | 83.2 |
| DM basis (%) |  |  |  |
| Crude protein | 24.7 | 22.4 | 10.8 |
| Crude fat | 20.9 | 3.7 | 1.1 |
| Ash | 5.6 | 5.1 | 8.0 |
| NDF | ND^2^ | 13.9 | 56.6 |
| ADF | ND | 7.3 | 33.7 |
| Starch | ND | 26.3 | 2.0 |
| Ca | 0.7 | 0.7 | 0.5 |
| P | 0.6 | 0.4 | 0.2 |
| ^1^Mixed hay composed of orchard and timothy hay.  ^2^ND = not determined. | | | |
