# Supplementary material for: Effects of Dietary Forage and Calf Starter Diet on Ruminal pH and Bacteria in Holstein Calves during Weaning Transition
Source: Front Microbiol. 2016 Oct 21;7:1575. doi: 10.3389/fmicb.2016.01575 (PMC5073099; doi:10.3389/fmicb.2016.01575)
Supplement: Supplementary Table S2 — Primer sequences used for qRT-PCR. [file Table2.DOCX]

**Supplementary Table S2. Primer sequences used for qRT-PCR.**

| Targets | Primer sequences (5'-3') | | Annealing Temperature (℃) | Efficiency | Reference |
| --- | --- | --- | --- | --- | --- |
|  |  |  |  |  |  |
| Total methanogens | F | GGTGGTGTMGGDTTCACMCARTA | 63 | 1.79 | Steinberg and Regan, 2008 |
|  | R | CGTTCATBGCGTAGTTVGGRTAGT |  |  |  |
| *Fibrobacter succinogenes* | F | GGTATGGGATGAGCTTGC | 60 | 1.88 | Tajima et al., 2001 |
|  | R | GCCTGCCCCTGAACTATC |  |  |  |
| *Selenomonas ruminantium* | F | TGCTAATACCGAATGTTG | 57 | 1.75 | Tajima et al., 2001 |
|  | R | TCCTGCACTCAAGAAAGA |  |  |  |
| *Ruminococcus albus* | F | CCCTAAAAAGCAGTCTTAGTTCG | 55 | 1.96 | Koike and Kobayashi, 2001 |
|  | R | CCTCCTTGCGGTTAGAACA |  |  |  |
| *Ruminococcus flavefaciens* | F | TCTGGAAACGGATGGTA | 55 | 1.79 | Koike and Kobayashi, 2001 |
|  | R | CCTTTAAGACAGGAGTTTACAA |  |  |  |
| *Streptococcus bovis* | F | CTAATACCGCATAACAGCAT | 57 | 1.61 | Tajima et al., 2001 |
|  | R | AGAAACTTCCTATCTCTAGG |  |  |  |
| *Megasphaera elsdenii* | F | GACCGAAACTGCGATGCTAGA | 58 | 1.98 | Ouwerker et al., 2002 |
|  | R | CGCCTCAGCGTCAGTTGTC |  |  |  |
